# “I'm Going to Do Everything I Can to Keep You From Having to Interact With the Police”: Queer Therapists' Perceptions of Police

**DOI:** 10.1111/jmft.70143

**Published:** 2026-05-11

**Authors:** Steph Cooke, Mary Cate Komoski, Lauren Jordan

**Affiliations:** ^1^ Relational Therapy Division Antioch University Seattle Washington USA; ^2^ Human Development and Family Science East Carolina University Greenville North Carolina USA

**Keywords:** crisis, police officers, queer, safety planning, therapists

## Abstract

This study investigated queer therapists' perceptions of police bias and attitudes toward police. We used a mixed‐methods explanatory approach by administering the Perceptions of Police Scale (POPS) to mental health professionals and conducted follow‐up qualitative interviews with participants who identify with a queer sexuality to explore the factors influencing their perceptions of police and the ways in which they view and utilize reliance on police when a client is experiencing a mental health crisis. Quantitative data were analyzed with SPSS using correlations and *t*‐tests to assess the relationship between sexuality and perceptions of police scores. Sexuality significantly correlated with perceptions of the police, meaning queer therapists viewed the police less favorably compared to heterosexual participants. A qualitative analysis using reflexive thematic analysis revealed seven themes that address how queer therapists perceive police and strategies utilized to decide if calling 911 for a client in crisis would be lifesaving or life‐threatening.

## 1 Introduction

Mental health crisis response in the United States today is shaped by systemic barriers that limit effective care. An estimated 20% of all 911 calls involve mental health crises (Frankham et al. 2021), which often result in police involvement. However, therapists have expressed concerns about the frequency and effectiveness of police response to mental health calls. Many of these concerns are centered around the ability for law enforcement officers to uphold procedural justice practices to calls that may be frustrating and beyond their area of expertise (Morgan 2024). Other concerns are in regard to the systematic and impersonal nature of these interactions between police and struggling individuals (Morgan 2024). This is alarming, given that law enforcement officers are becoming increasingly utilized in crisis interventions relating to mental health (Kuehl et al. 2023). The overall connotations of police as punitive also serve to complicate the effectiveness of their involvement in such sensitive situations.

Additionally, recent studies have shown that many officers feel underprepared and undertrained to respond to these calls in an effective manner (Kuehl et al. 2023). Concerns over interagency collaboration are also making their way into discussions. As many as 89% of officers in Kuehl et al. (2023) study suggested that getting the necessary support from mental health professionals was their biggest challenge in crisis calls. Several officers felt that mental health professionals should be the ones responding to mental health crisis calls (Kuehl et al. 2023). Regardless of these research findings, the police are still the most visible resource and the one that therapists are often trained to rely on. This places therapists in an ethical dilemma. Therapists must prioritize the safety of their clients, which may mean considering the potential risks of police involvement.

### 1.1 Historical Context of Social Location and Systemic Oppression

Systemic oppression is defined as the social stratification that arises when power and resources are concentrated in dominant groups at the expense of others (Copeland and Kilgo 2024). Individuals with less social power and limited access to resources are pushed to the margins of society and experience discrimination, stigmatization, and the internalization of negative societal perceptions due to their social location, all of which can have profound impacts on mental health (Meyer 2003). Within this broader context, the LGBTQ+ community has been disproportionately subjected to police surveillance, victimization, and targeting. One of the most notable instances is the 1969 Stonewall raid and subsequent riots. Police frequently targeted LGBTQ+ spaces and used entrapment strategies to arrest these individuals for alleged violations (Siodmak 2018). While Stonewall served as a catalyst for the gay rights movement, it did not stop victimization from the police; rather, the profiling evolved into different forms. Those in the LGBTQ+ community were subjected to the unjust applications of sodomy laws that were used to penalize consensual same‐sex sexual encounters (Jones and Maguire 1999). It was not until Lawrence v. Texas (2003) that the overturning of these law applications took place. In the decades since, LGBTQ+ folks have continued to resist oppressive laws through struggles for marriage equality, access to affirming healthcare, protection and respect as athletes, and inclusive curriculum in schools. Together, these examples illustrate the ongoing targeting of LGBTQ+ individuals.

### 1.2 Learning From Queer Therapists' Crisis Practices

Literature reflects these historical and ongoing patterns of discrimination and harm, suggesting that LGBTQ+ individuals are more likely to have negative experiences with law enforcement compared to straight individuals (American Civil Liberties Union 2024; Owen et al. 2018), though how this shapes professional attitudes toward police intervention remains underexplored. LGBTQ+ (i.e., queer) therapists, informed by these lived experiences, may be more inclined to explore non‐police alternatives when supporting clients in crisis. This tendency is significant because clients who also hold marginalized identities may face reduced risk in interventions that do not involve law enforcement (Davis et al. 2025; Marcus and Stergiopoulos 2022). Understanding how queer therapists navigate these decisions offers valuable insight for the broader mental health field. Their practices may offer critical insight: What strategies do they employ to work around systemic reliance on police? How do these approaches serve clients' safety and trust? And what can other providers learn from these adaptations? Examining these questions can inform the development of crisis response protocols that prioritize equity, minimize harm, and reflect the realities of marginalized communities.

### 1.3 Theoretical Framework

Meyer (2003) introduced the minority stress model to help explain the social, psychological, and structural influences on sexual minorities' mental health and well‐being. Excess exposure to distal and proximal stressors underscores this framework (Frost and Meyer 2023). Meyer (2003) described distal stressors as external forces (e.g., people or institutions) that uphold discriminatory practices specifically affecting the well‐being of sexual minorities, which could include microaggressions, poverty, and a loss of income. Proximal stressors are the internal experiences of self‐rejection and the expectation that others will reject them because of their identity as a sexual minority (Meyer 2003). These are not entirely mutually exclusive processes; rather, the fear of distal stressors can lead sexual minorities to conceal their identity (Pachankis et al. 2020). Concealment is a form of self‐protection for some, but it's also an isolating experience that limits access to necessary support and care.

According to minority stress theory, stress stemming from stigmatization of one's identity leads to an increase in emotional distress and dysregulation (Sarno et al. 2020). Research consistently shows that LGBTQ+ individuals report higher levels of mental health challenges than their non‐LGBTQ+ peers, including elevated risk for suicide and suicidal ideation (Cochran et al. 2003). However, the lack of “institutional investments” needed to adequately support these individuals must also be examined alongside individual responses to distal and proximal stress (Meyer 2014, 349). Investments in equitable and trustworthy crisis response options are one pathway to reducing the stress that minorities experience. From a provider standpoint, there is scant research examining how queer therapists navigate reliance on federal and local institutions (e.g., law enforcement, calling 911) and whether they perceive these systems as *socially safe* for their clients to rely on as well. Social safety is compromised by discrimination, prejudice, and stigma (Frost and Meyer 2023). Thus, minority stress theory is a relevant framework for challenging the field's current understanding of mental health crisis response, and queer therapists are uniquely positioned to provide key insights.

## 2 Study Purpose

Two research questions guided the study to examine therapists' views and decision‐making related to police involvement in mental health crises: (1) What are therapists' perceptions of police? and (2) How do therapists describe the experience of navigating police involvement in responding to a mental health crisis? To address these questions, the study investigated the relationship between therapists' sexual identities and their attitudes toward police, and explored whether therapists' experiences of their queer identities (e.g., lesbian, gay, bisexual, etc.) influence their reliance on police during mental health crisis response. To achieve this, the authors used a mixed‐methods design that allowed them to examine patterns quantitatively and then explore their meaning qualitatively.

Understanding the intersection of how therapists identify themselves socially, their perceptions of police, and how therapists navigate the involvement of police in the context of client mental health emergencies is essential for safety planning, training culturally aware therapists and supervisors, and developing the critical consciousness necessary to understand the anticipated negative impact of police presence in clinical settings. This study is especially relevant for therapists who have been pushed to the margins in society due to discrimination or prejudice because of how they identify or how society perceives them. Although this study initially aimed to examine multiple marginalized identities, only sexual identity (LGBQ) demonstrated significant differences in perceptions of police. Therefore, the present manuscript focuses on therapists who identify as LGBQ. Queer, an umbrella term in the LGBTQ+ community encompassing individuals who are not heterosexual and/or cisgender, will be used throughout the remainder of this paper. However, eligibility for the qualitative interviews required participants to identify only as a sexual minority to be eligible; gender identity was not included as an inclusion criterion. Potential explanations for the lack of statistical significance among other identity groups are mentioned in Section 7.

## 3 Methods

An explanatory mixed‐methods design was utilized to collect quantitative data on therapists' perceptions of police bias and attitudes toward police, followed by a qualitative study to expand on the results of the survey. Mixed‐methods integration occurred at two points: (1) during sampling, where qualitative participants were selected based on quantitative results (queer identity, high POPS scores), and (2) during interpretation, where qualitative themes were used to contextualize and explain significant quantitative findings. Study procedures were approved by the Institutional Review Board (IRB) at East Carolina University. All participants who participated in the entire study consented to completing the Qualtrics survey and one individual interview via Microsoft Teams, led by the first author.

A purposive sampling method was used to recruit survey participants. This study targeted a specific subset of mental health professionals, resulting in a more focused aim that yielded rich qualitative data from interviews with eight participants, thereby supporting the appropriateness of a smaller data set (Braun and Clarke 2022). Flyers were shared on online platforms such as Instagram and Facebook, which provided information about the study, IRB number, and the first author's contact information. At the time of the study, the flyer was also posted on the first author's office door, located in a university building that also housed a Couple and Family Therapy program. People with access to the flyer online or in person were encouraged to share with their professional networks. Participants who completed the individual interview were recruited via email when they were given the option on the survey to have the first author follow up with them. These participants had to finish the entirety of the survey, identify as queer, and report having negative perceptions of police to stay within the scope of the qualitative component of the study, as determined by the literature review, guiding theory, and survey results.

Confidentiality of participants was maintained through de‐identification of all participant data, including the removal of potentially identifying information on interview transcripts related to participants' professional roles and affiliations, as well as contextual information regarding specific geographic areas (e.g., neighborhoods) and names of local establishments (e.g., hospitals). Additional safeguards included secure storage of audio recordings and transcripts on IRB‐approved, password‐protected devices and limiting data access to the research team. Extra care was taken at the conclusion of each interview to invite participants to reflect on their emotional experience of the questions asked. This debriefing practice helped to ensure participants felt settled enough to leave the interview and transition back to their daily lives. Given the first author's training and qualifications as a licensed marriage and family therapist, they felt confident in their ability to debrief with each interview participant and did not observe any significant emotional responses that required additional resourcing or support.

### 3.1 Inclusion and Exclusion Criteria

The quantitative portion of the study had four inclusion criteria. Participants had to be over the age of 18 and fully or provisionally licensed as a therapist or counselor. There were language and geographical restrictions, such as participants had to be English‐speaking and currently residing in the United States. If eligible, participants were required to understand the nature of the study by completing the informed consent, with an electronic signature, via Qualtrics. After the researchers analyzed the quantitative results indicating significant differences between queer and non‐queer therapists regarding perceptions of police, the qualitative portion of the study had two additional criteria: participants had to have scores that indicated less favorable views of police and identify as a sexual minority. The results from the quantitative analysis revealed only a significant relationship between therapists' perceptions of police and sexual identity, compared to racial identity and gender identity. Thus, to stay within the scope of the current study, the researchers did not base their inclusion criteria on whether a participant identified as transgender, cisgender, white, or a person of color.

### 3.2 Qualitative Interview Protocol

The first author conducted qualitative interviews with eligible participants. This author is a trained and published qualitative researcher, demonstrating their adequate preparation to conduct qualitative interviews. Participants were made aware that, by doing this interview, the researchers hoped to learn more about the factors influencing queer therapists' perceptions of police and the ways in which they viewed and utilized police reliance when a client experienced a mental health crisis. For example, participants were asked, “What specific examples from your lived experience have influenced your views on police? How does your identity as a LGBTQ+ person shape those experiences?” Additional questions about their experience of crisis response and police reliance included, “What do you consider some of the challenges a therapist or counselor might face when responding to a mental health crisis? How does your LGBTQ+ identity shape your perception of these challenges? In your profession, how do you assess and respond to a client experiencing a mental health crisis? To what extent have you involved the police in responding to a mental health crisis your client was experiencing? Which aspects were positive? Negative?” If participants reported no prior reliance on police, the interviewer probed for additional context to better understand their decision‐making. Interview participants chose a pseudonym during the interview, and all of their documents were renamed directly after the interview ended to protect their identity.

### 3.3 Quantitative Demographics

A total of 92 participants were included in the final quantitative analysis. A detailed breakdown of gender, sexuality, and race is presented in Table 1.

**Table 1:** Demographic characteristics of the sample.

Baseline characteristic	LGBQ		Non‐LGBQ		Full sample	
	*n*	%	*n*	%	*n*	%
Gender						
Agender	1	2.5	0	0	1	1.1
Cis man	3	7.5	9	17.3	12	13.0
Cis woman	27	67.5	42	80.8	69	75.0
Non‐binary	8	20.0	0	0	8	8.7
Trans woman	1	2.5	1	1.9	2	2.2
Sexuality						
Another sexual orientation	1	2.5	0	0	1	1.1
Asexual	1	2.5	0	0	1	1.1
Bisexual	11	27.5	0	0	11	12.0
Gay	3	7.5	0	0	3	3.3
Heterosexual or Straight	0	0	52	100	52	56.5
Lesbian	8	20.0	0	0	8	8.7
Pansexual	5	12.5	0	0	5	5.4
Queer	10	25.0	0	0	10	10.9
Questioning	1	2.5	0	0	1	1.1
Race/Ethnicity						
Asian or Pacific Islander	1	2.5	1	1.9	2	2.2
Black or African American	4	10.0	9	17.3	13	14.1
Hispanic or Latino	1	2.5	4	7.7	5	5.4
Multiracial or Biracial	2	5.0	1	1.9	3	3.3
White or Caucasian	32	80.0	37	71.2	69	75.0

The majority of participants identified as cisgender, with 69 identifying as cisgender women and 12 as cisgender men. In terms of sexual orientation, 52 participants identified as heterosexual or straight, followed by 11 identifying as bisexual and 10 as queer. Individuals who identified as heterosexual or straight were coded as “0” and all other responses were coded as “1” for the purpose of analysis. Sexual identity was dichotomized as heterosexual versus sexual minority (all non‐heterosexual identities) due to small sample sizes within individual sexual identity groups, which limited statistical power for meaningful subgroup analyses. Collapsing categories allowed for more stable estimates and reduced the risk of underpowered comparisons. This approach is also theoretically aligned with minority stress theory, which emphasizes structural differences between heterosexual individuals and sexual minority populations in exposure to stigma and institutional bias. Nonetheless, this analytic decision necessarily obscures within‐group variability among sexual minority identities and should be addressed in future research with larger, more diverse samples.

Regarding racial identity, 69 participants identified as White or Caucasian, while Black or African American was the second most common response, reported by 13 participants. Gender was assessed using a forced‐choice, single‐response format, with response options listed in Table 1. Sexual orientation was also collected using a forced‐choice format, with an “other” option allowing for write‐in responses. Race and ethnicity were assessed using two formats: a “select all that apply” question to capture participants' full racial/ethnic identity and a “select the one that best applies” question, which was used for analysis. Data from the second question are presented in Table 1. This dual approach aligns with best practices for balancing identity complexity with analytical clarity (Sharghi et al. 2024).

### 3.4 Qualitative Demographics

A total of eight therapists completed the semi‐structured qualitative interview. All eight participants were sexual minorities (see Table 2 for a summary of the demographics). Participants identified sexually as queer (*n* = 3), bisexual (*n* = 3), pansexual (*n* = 1), and demisexual lesbian (*n* = 1). We use the term queer throughout the findings to be inclusive of non‐heterosexual sexual identities. The majority of participants identified racially as white, with one participant including Hispanic/Latino for their ethnicity. Most of the participants (*n* = 5) identified as cis women, with the remaining participants identifying as non‐binary (*n* = 2) and agender (*n* = 1). The clinicians interviewed were Marriage and Family Therapists (*n* = 3), Clinical Social Workers (*n* = 2), a Clinical Mental Health Counselor (*n* = 1), a Clinical Addiction Specialist (*n* = 1), and a Psychologist (*n* = 1). The current work setting of the participants helped to contextualize their responses, with most working in a private practice (*n* = 6), and the remaining two clinicians currently working in a community mental health agency (*n* = 1) and a university/academic setting (*n* = 1).

**Table 2:** Demographic characteristics of interview participants.

Pseudonym	Sexual identity	Race/Ethnicity	Gender identity	Professional credential	Work setting
Andrea	Bisexual	White	Cis woman	Licensed Clinical Social Worker	Private practice
Elizabeth	Pansexual	White	Cis woman	Licensed Psychologist	Private practice
Indigo	Bisexual	White	Cis woman	Licensed Clinical Mental Health Counselor Associate	Private practice
Jake	Queer	White	Non‐binary	Licensed Marriage and Family Therapist	Private practice
Katie	Demisexual lesbian	White	Cis woman	Licensed Clinical Addiction Specialist	Community mental health agency
Marie	Queer	White, Hispanic/Latino	Non‐binary	Licensed Marriage and Family Therapist	Private practice
Mary	Queer	White	Cis woman	Licensed Clinical Social Worker	Private practice
Willow	Bisexual	White	Agender	Licensed Marriage and Family Therapist Associate	University/Academic setting

## 4 Measures

### 4.1 Perceptions of Police Scale (POPS)

The Perceptions of Police Scale (POPS), developed by Nadal and Davidoff (2015), is a 12‐item measure assessing individuals' attitudes toward police. The scale is comprised of two subscales: General Attitudes Toward Police (9 items) and Perceptions of Bias (3 items). Sample items for General Attitudes Towards Police include “Police officers care about my community,” and “The police provide safety.” Sample items for Perceptions of Bias include “Police officers treat all people fairly,” and “The police do not discriminate.” Items are rated on a 5‐point Likert scale, with 1 indicating “I strongly agree” and 5 indicating “I strongly disagree.” Thus, higher scores indicate less favorable views of the police.

Nadal and Davidoff (2015) established the scale's reliability, reporting strong internal consistency with Cronbach's alpha coefficients of 0.93 for the General Attitudes Toward Police subscale, 0.88 for the Perceptions of Bias subscale, and 0.94 for the total scale. The scale authors also determined construct validity based on qualitative data collected during pilot testing. This scale was selected for the study because of its niche content, strong reported reliability, and concise nature.

## 5 Data Analysis

### 5.1 Quantitative Analysis

The quantitative component employed a cross‐sectional survey design using the Perceptions of Police Scale (POPS). Data were analyzed using the Statistical Package for the Social Sciences (SPSS, Version 29; IBM Corp (2023), Armonk, NY). Descriptive statistics and bivariate correlations were computed to examine the dataset. Cohen's (2013) criteria were used to interpret correlation effect sizes, with *r* = 0.30 indicating a moderate effect and *r* = 0.50 indicating a strong effect size.

Given significant correlations, independent‐samples *t*‐tests were conducted to compare group means between those who identified as LGBQ and those who did not. Levene's test for equality of variances indicated a significant difference in variance between the two groups for the Bias Towards Police subscale, necessitating the use of Welch's *t*‐test for that analysis. For the Attitudes Towards Police subscale and the total Perceptions of Police Scale score, equal variances were assumed.

### 5.2 Qualitative Analysis

The first and second authors served as coders for this study and followed Braun and Clarke's (2022) reflexive thematic analysis guidelines to: (a) familiarize the data; (b) code the data; and (c) define, name, and refine themes. This process was iterative in nature to comprehensively address each of the research questions. Transcripts from the interview were analyzed using a qualitative coding software, NVivo, to track similarities across responses, specifically highlighting patterns in participants' experiences (Braun and Clarke 2022; Lumivero 2025). The coders read each transcript closely, noting relevant quotes that answered the research questions (Braun and Clarke 2022). Each coder independently completed a first round of coding for each transcript. The coders reviewed the coding together periodically throughout the process, meeting at least three times to review their codes. Insights were shared and interrogated by the coders, and themes were created. Thematic sufficiency was guided by analytic depth and was considered complete once a comprehensive understanding of the research questions was attained by the coders. As themes were developed, the coders analyzed cases that contradicted preliminary themes and returned to the qualitative transcripts to reinterpret coding and refine thematic decisions. Conclusions were drawn to discern how queer therapists described their experiences with, and perceptions of, police within and outside the context of therapy.

### 5.3 Reflexivity and Rigor

The research team consisted of two PhD‐level academics, one of whom is a trained systemic therapist, and a student. The first author is a licensed marriage and family therapist and was a postdoctoral researcher at the time the data were collected. Their experience as a Black, genderfluid, queer clinician and academic with expertise in crisis safety planning informed the research questions and interview guide. The second author is a white, heterosexual, cisgender woman who works as a professor at a public Southeastern university. Her work outside of this project explores how people think about trauma, offering some insight into analyzing how individuals may process emotionally‐charged interactions with police. The third author is a white, heterosexual, cisgender woman who was an undergraduate research assistant during the early stages of this study with a strong interest in inclusive crisis response. Experience and interests combined, the research team brought critical insights to the research. To ensure reflexive analysis, the first and second authors met throughout the analysis and write‐up of the study's findings to discuss their assumptions and perspectives of the participant responses, codes, and emerging themes. These ongoing dialogues created intentional spaces to question ideas and examine how each author's social location informed their meaning‐making. The authors also shared excerpts from analytic memos created during coding, which captured the first and second authors' emotional reactions to the transcripts and their analytic insights. This collaborative and reflexive process deepened the authors' understanding of the data and helped to manage bias; thus, ensuring a thoughtfully interrogated analysis (Braun and Clarke 2022).

## 6 Results

**Figure 1:**
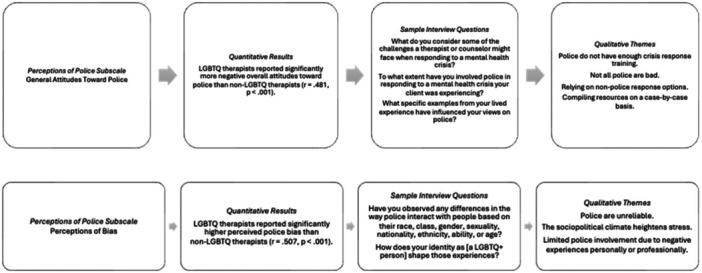
Mixed‑methods findings on therapist perceptions of police.

The purpose of the study was to understand how LGBQ (i.e., queer) therapists perceive police bias and their general attitudes toward police. The following section details the results from the Perceptions of Police Scale (POPS) (Nadal and Davidoff 2015) and findings from the qualitative analysis using Braun and Clarke's (2022) reflexive thematic analysis to help explain and build on the survey results by including questions about police reliance in crisis response (see Figure 1).

### 6.1 Quantitative Results

Descriptive statistics of the Perceptions of Police Scale and its subscales are included in Table 3. Scores on the total scale and its subscales all represented strong reliability, with Cronbach's alphas ranging from 0.85 to 0.96. Data were generally normal, as measured by a Shapiro–Wilks test, though the Perceptions of Bias subscale showed significant deviance from normality. Independent‐samples *t*‐tests are generally robust to moderate violations of normality assumptions, particularly when group sizes are reasonably balanced, and effect sizes are large, as observed in the present study.

**Table 3:** Descriptive statistics for Perceptions of Police Scale.

Variable	No. of items	Mean	Std. dev.	Cronbach's alpha	Shapiro–Wilks
Perceptions of Police	12	41.07	10.76	0.96	0.98 (*p* = 0.102)
General Attitudes Toward Police	9	3.25	0.92	0.95	0.98 (*p* = 0.152)
Perceptions of Bias	3	3.95	0.92	0.85	0.90 (*p* < 0.001)

Results of correlation analysis are presented in Table 4. A large, positive, significant correlation was found between sexuality and both the Attitudes Toward Police and Bias Toward Police subscales. Additionally, the total POPS score was significantly correlated with sexuality, indicating that sexuality is associated with both the overall construct and its specific components. The high correlation between the Perceptions of Police Scale total scores and its subscales is expected due to scale construction.

**Table 4:** Pearson correlation between sexuality and the Perceptions of Police Scale.

Variable	*n*	*M*	SD	1	2	3	4
1. Sexuality	92	0.43	0.498	—			
2. Attitudes Towards Police	92	3.25	0.92	0.481**	—		
3. Perceptions of Bias	92	3.95	0.92	0.507**	0.857**	—	
4. Total Perceptions of Police	92	41.07	10.76	0.501**	0.991**	0.918**	—

** *p* < 0.01.

Results of *t*‐tests comparing LGBQ and non‐LGBQ participants on the Perception of Police scale are included in Table 5. LGBQ participants reported significantly higher scores on the scale, indicating more negative Attitudes Towards Police and greater perceptions of police being biased than non‐LGBQ participants. These differences were not only statistically significant but also represented very large effect sizes, suggesting meaningful implications for crisis response planning.

**Table 5:** Independent *t*‐tests for LGBQ and non‐LGBQ participants on the Perception of Police Scale.

Outcome variable	Non‐LGBQ		LGBQ		*t*(90)	*p*	Cohen's *d*
	*M*	SD	*M*	SD			
Total Perceptions of Police	36.37	9.41	47.18	9.30	−5.49	< 0.001	−1.60
Attitudes Towards Police	2.86	0.80	3.75	0.83	−5.20	< 0.001	−1.09
Perceptions of Bias	3.54	0.84	4.48	0.75	−5.67	< 0.001	−1.17

### 6.2 Qualitative Findings

In total, seven themes emerged from the data. The main themes were organized according to the two research questions. We identified four themes for the first research question, “What are therapists' perception of police?” (a) police are unreliable; (b) police do not have enough crisis response training; (c) the sociopolitical climate heightens stress; (d) not all police are bad. We identified three themes for the second research question, “How do therapists describe the experience of navigating police involvement in responding to a mental health crisis?” (a) limited police involvement due to negative experiences personally or professionally; (b) relying on non‐police response options; (c) compiling resources on a case‐by‐case basis. The following section will include descriptions of each theme. Themes are informed by minority stress theory with exemplary quotes that support the main themes and theoretical concepts.

#### 6.2.1 Police Are Unreliable

Queer therapists described police as being inconsistent in how they approached crisis response, which positioned police as being unreliable among participants. The lack of continuity in helpfulness led queer therapists to question if police can be a resource they relied on when their clients experienced a crisis. For example, Willow described police officers as “unreliable” because “I call a police officer and they're helpful, and sometimes I call a police officer and they're terrible and rude and prejudiced. I think probably more often prejudice than not.” Willow described a tension inherent in their decision‐making that illustrates the interplay between distal stressors (e.g., ongoing experiences of prejudice) and proximal stressors (e.g., expectations that police would be unhelpful), consistent with previous research demonstrating how stigmatized groups often experience negative treatment by institutional authorities (Meyer 2003). Katie echoed this sentiment of “not being sure who's going to show up, with what different biases… I think sometimes things can either get pushed off or seen as unimportant, or I think things could escalate in a dramatic way. I think it tends to be one or the other.”

#### 6.2.2 Police Do Not Have Enough Crisis Response Training

Personal and vicarious experiences of cops and their interactions with people in emotional distress informed queer therapists' understanding of potential biases that their clients may face should 911 be called. Participants were less concerned with convincing clients that cops are either *good* or *bad* and instead cared more about centering the client's perception and possible concerns about police interactions if they call 911 for support. The concerns that queer therapists raised relate to the perceived lack of training that police officers have to respond to a mental health emergency. From a minority stress perspective, queer therapists' expectations of inadequate crisis response from police officers are realistic and informed by chronic exposure to narratives of discrimination and marginalization (Meyer 2003), specifically in interactions with law enforcement. When considering the added factor of a client's social location, Elizabeth said, “From what I hear, especially from my crisis folks, in any crisis, they do not want to involve the police, especially if they're a member of any marginalized community, race, LGBTQ, whatever it might be, poverty, people who are really poor.”

#### 6.2.3 The Sociopolitical Climate Heightens Stress

Descriptions of heightened stress within the current sociopolitical climate reflect repeated exposure to structural stigma (Meyer 2003), which is characterized as political discourse and hostility toward minority groups. Participants explored their reactions to coverage of police officers in the media, the rights of minoritized populations (e.g., LGBTQ+ and people of color), and police propaganda. Marie elaborated on media coverage of police officers by sharing, “I've seen that happen in plenty of videos… They say something and the person doesn't understand and they just escalate the situation by yelling it louder, rather than trying to clearly explain what is going on. So just anxiety and not trustworthiness around that.” For queer therapists, a tense sociopolitical climate functions as a significant minority stressor that perpetuates the ongoing psychological burden of navigating police involvement. Katie discussed the implications of negative political discourse on their perception of police: “I think looking at just politics as a whole, there's such an ugly push around queer stuff in politics, and that directly impacts how police are going to interact with people, which is already a questionable thing, especially depending on what region you live in.”

#### 6.2.4 Not All Police Are Bad

Participants described exceptions to the negative experiences they had with police when prompted to identify any positive interactions they had. Andrea disclosed, “Police have resources I don't have. Police have training in restraining people. They're going to have first aid training. They have access to training that I don't have. And so that would be a benefit.” From Mary's viewpoint, police do help some people, people who “have white skin and people who have a lot of income… The police do try to help.” Similarly, Marie reported, “I try really hard to continue to have at least a neutral view towards the police because I really do want to fundamentally believe that not all of them are awful… I know they do help some people, just not all people.” Queer therapists describe an awareness that police are helpful if clients assume a level of privilege along the lines of race, gender, sexuality, and socioeconomic status, to name a few; however, police may be less helpful if you occupy a less privileged position in society. Previous research supports this complicated intersection between social location and perceptions toward police (Owen et al. 2018).

#### 6.2.5 Limited Police Involvement Due to Negative Experiences Personally or Professionally

In deciding to what extent to involve police in crisis response interventions, queer therapists drew from both personal and professional interactions with law enforcement. Encounters in which they or their clients experienced or witnessed stigma, prejudice, and discrimination related to identity or mental health contributed to stressful and often harmful social interactions with police officers. For example, Marie recounted an encounter they had with police at a young age, “I was walking home from the bus stop, and my family lived in a more affluent area… There was a police officer who rolled up on me as I was walking home from the bus stop and asked me what I was doing there. And I was in second or third grade, so I was maybe nine. And just that anxiety of, ‘Well, what am I doing here? I'm going home, I know what I'm doing here,’ but feeling like I was wrong, and it being framed in that I was wrong.” Regarding professional experiences, Mary reflected on their experiences with police as a young social worker, “They would assume that I was trying to buy drugs 'cause I was a young white woman and not actually there as a professional going to help a client. [I'd] pick her up so we could go get her signed up for food stamps, and get her diapers, and get her WIC, and get her signed up for childcare vouchers. And so, they would pull me over.” Similarly, Jake expressed concerns about their professional experiences with police during a crisis when 911 was called, “[police] are talking about the client while they're two feet away really poorly, making fun of mental health, essentially. Like while a kid's breaking down, they're like, ‘oh, kids and their drama.’ So belittling… Making it worse.” The social stress of negative police encounters can significantly shape queer therapists' lives, reinforcing perceptions of police bias and negative attitudes toward police officers (Meyer 2003).

#### 6.2.6 Relying on Non‐Police Response Options

Queer therapists described barriers or challenges to crisis response, referenced in quotes from previous themes, which led most of them to not want to rely on 911 for crisis response. According to the minority stress model, queer therapists' reliance on non‐police crisis options reflects the “adaptation effort” or added physical and emotional labor required of stigmatized groups compared to non‐stigmatized groups to cope with potential discrimination (Meyer 2003, 4). Participants described making intentional adjustments to reduce the anticipated risks associated with police involvement while they continued to deliver effective, ethical care to clients experiencing a crisis. For example, Willow shared how they decenter the use of police in their roles as a therapist and educator, “I never recommend that a client calls 911, and in fact, I taught a class the other day where someone asked about it and I said, you need to be thinking about your client, and is it safe for them to call 911? I've never recommended to a client to call 911, honestly. I've always recommended that they call the mental health or mobile crisis situation wherever I am, or go to the emergency room where a nurse and social worker will probably be available, or a psychiatric professional will probably be available, even if they have to wait 3 h.” Involving other mental health professionals, and even close friends or family members, in the crisis response process was important. Elizabeth shared, “I try to avoid ever calling the police. But if it's imminent, if the person says, ‘I'm going to leave here and shoot myself,’ then I still try to do other things, but then you do what you have to do. Mobile crisis is a good one if they're at home. But usually, I can get a friend or family member to help. Usually, the severity of the symptoms subsides. So, I try to avoid the police, calling emergency services, whenever I can.”

#### 6.2.7 Compiling Resources on a Case‐by‐Case Basis

Determining the best course of action to follow relied on several factors, including how severe the clients' crisis was, the resources available, the therapists' connection to resources and other mental health professionals, the age of the client, and the clients' support system, to name a few. Willow shared, “I ask a series of questions, which I think probably most therapists do to sort of gauge, okay, how serious is this? Is it truly a crisis or is the client just feeling super upset? And then what I've historically done is, if it is a true mental health crisis, I've never called the police… I have always tried to just involve the parents.” Ultimately, participants described a collaborative decision‐making process that avoided the use of police and varied case to case. Indigo reported, “And because of my past experience work wise, I have lots of connections where I can reach out and be like, ‘Hey, I have this going on.’ And actually, that's probably something to my advantage as well. I can do direct admissions sometimes depending on the case. So that makes a difference too. I feel like there needs to be a better network in the system to potentially be able to avoid having to call the police.” The continuum of distal and proximal stressors that queer therapists navigated emerged from external stressors (e.g., policies and institutions beyond the individual) and internal stressors (e.g., personal meanings or expectations based on their experiences) (Meyer 2003). Taken together, these layered stressors helped explain why queer therapists remained vigilant in their attempts to keep clients safe from potentially life‐threatening police encounters, making decisions that reflected the resources and support available, even when presented with limited options. As Mary stated, queer therapists tried, “giving [clients] a choice… to help people feel like they might have some options even if they don't have that many.”

## 7 Discussion

This study employed an explanatory mixed‐methods design to capture both the breadth and depth of therapists' perceptions of police. Quantitative findings established significant differences in attitudes toward police based on sexuality, while qualitative themes provided context for these patterns using the minority stress model as a conceptual framework to explain how aspects like lived experiences and sociopolitical factors shape clinical decision‐making.

The magnitude of the differences in perceptions of police between LGBQ and non‐LGBQ therapists is noteworthy. Cohen's *d* values exceeded 1.0 across all measures, indicating very large effect sizes and suggesting that sexual identity is a robust predictor of attitudes toward police. These findings reflect prior literature, which demonstrates that marginalized individuals are more likely to experience police encounters, especially negative experiences (American Civil Liberties Union 2024; Girardi 2022). The absence of significant differences by race or gender may reflect the sample's demographic composition (predominantly White, cisgender) or suggest that sexuality is a more salient factor in shaping these attitudes. Taken together, these results underscore the need to learn from therapists of various social locations and lived experiences in refining crisis response protocols.

Importantly, these differences do not exist in isolation—they are symptoms of and intersect with broader issues in the mental health system. Relying on law enforcement for mental health emergencies is a flawed resource, especially when the social location and concerns of therapists and clients are considered. Therapists in this study discussed the perceived lack of training that police officers are provided with to effectively de‐escalate mental health emergencies with people who hold fewer social privileges due to stereotypes, prejudice, and discrimination (Kuehl et al. 2023; Morgan 2024). Unreliable or insufficiently trained police pose serious risks for clients in crisis. Safety planning practices, while intended to reduce suicide risk (Nuij et al. 2021), can rely on law enforcement in ways that undermine client trust, especially for individuals who may experience state systems as unsafe (Taylor and Kuo 2019; Wortzel et al. 2019).

The distrust and fear of police being unhelpful has also left many therapists and clients with limited options in a crisis situation (Morgan 2024; Owen et al. 2018). Given the ongoing distrust of police by marginalized communities, alternative response programs are being implemented in several countries (Bahr et al. 2025; Marcus and Stergiopoulos 2022). Non‐police response teams are designed to handle mental health emergencies that do not rely on police officers and instead include professionals such as social workers, licensed clinicians, and trained civilians (Watson et al. 2025). Therapists in this study utilized non‐police options such as mobile crisis units and family members, when available.

Queer therapists' decision‐making toward police involvement included distal and proximal stressors consistent with minority stress theory (Meyer 2003). Experiences of and exposure to discrimination, structural stigma within the current sociopolitical climate, and prior negative interactions with law enforcement shaped realistic expectations that police responses may be inadequate or unsafe. These chronic stressors, including internalized expectations of bias and risk, contributed to the heightened vigilance and ongoing psychological toll of assessing the potential consequences of police involvement. As a result, participants frequently engaged in adaptation efforts (Meyer 2003) that prioritized non‐police crisis responses, reflecting the additional labor required of queer therapists to protect their clients while still providing effective care. Collectively, these processes help explain why queer therapists deliberately sought alternatives and remained cautious in their clinical decision‐making.

Furthermore, the findings of this study underscore the importance of supporting queer therapists in ethical decision‐making practices. Queer therapists in this study exercised their clinical judgment on a case‐by‐case basis, which took into account the unique needs of a client in crisis. Tailoring care to each client in need may increase the intensity and frequency of queer therapists experiencing burnout from their job as mental health providers. Previous studies have suggested that sexual minority mental health practitioners experience greater burnout and lack of support compared to their heterosexual counterparts (Viehl et al. 2017). When queer therapists feel supported, without contemplating the lack of client‐centered and inclusive resources available, they might be better equipped with the crisis interventions needed to prevent exposing clients to potentially life‐threatening encounters with police.

### 7.1 Clinical Implications

Input from queer therapists has important implications for therapists working with clients in crisis. Results of this study suggest their awareness of social location and systemic barriers can inform approaches that benefit anyone with a marginalized identity. More broadly, mental health professionals need to prioritize ongoing dialogues with clients, supervisors, supervisees, and colleagues about informed consent and safety planning procedures that create life‐affirming alternatives, rely on networks of support, and center the agency of the client (Drustrup et al. 2023). It is imperative that educators and supervisors engage in critical conversations about the potential impact of police presence on clients who have had negative or traumatic encounters with law enforcement in their communities. These discussions can take place in graduate training programs and as part of continuing education (CE) requirements dedicated to ethics.

Based on the findings from this study, it is critical for educators and supervisors to understand that some licensed therapists or therapists‐in‐training may hold unfavorable views of police or perceive police as biased. The following bulleted list provides discussion questions to help facilitate discussions with other therapists, students, and supervisees:

- What level of comfort do you experience in the presence of police? What specific behaviors by police officers increase or decrease your sense of comfort?
- What police‐involved crisis response options are available in the state where you are licensed or are considering licensure? What mental health crisis intervention training do these police officers receive?
- On a scale from 0 to 10, with higher scores indicating greater confidence, how confident are you that police will respond effectively to a mental health crisis? What factors influence your rating, and what keeps it from being higher or lower?
- How do your own observations and encounters with police shape your perceptions of police effectiveness?
- What non‐police crisis response alternatives are available in the state where you are licensed or are considering licensure?
- How do you, or how will you, determine whether a crisis response resource is safe and effective for your client to rely on?

As our study revealed, therapists' own negative encounters with or assumptions about police had an impact on their decision‐making. Opportunities need to be provided for mental health professionals to self‐reflect on their own comfort with police, develop familiarity with the extent of police involvement in crisis response calls within their county or state, and engage in a follow‐up activity to identify credible non‐police crisis response alternatives. These discussions can better equip professionals with the safety resources available to them and their clients. Mental health professionals with limited crisis response options available to their clients may also benefit from contacting their local police departments and inquiring about mental health crisis intervention training and mental health referral protocols. If 911 is called, having a clear plan in place that prioritizes the therapist's clinical judgment and concerns of the client could be life‐saving.

### 7.2 Limitations and Future Directions

Though this study made several contributions to the literature on inclusive mental health crisis response, there were limitations to this research. Although group sizes were modest, the consistently large observed effect sizes provide some confidence in the statistical power for the primary comparisons between LGBQ and non‑LGBQ participants; however, the sample size limited statistical power to examine subgroup differences within sexual minority identities or to test interaction effects across social identities. An additional limitation was the exclusion of participants from the qualitative interviews who reported favorable views of the police on the survey. While this decision maintained a theoretically and literature‐informed investigation into perceptions of police bias and attitudes toward police among a socially marginalized group, it may have limited the range of perspectives represented and the transferability of the qualitative findings. Self‐selection bias may also help to explain the difference between non‐participants and individuals who volunteered to complete the survey and interview (Alarie and Lupien 2021). This study would benefit from a more diverse sample represented in the qualitative interviews, specifically examining how race, ethnicity, age, geographic location, and socioeconomic status shape positive, negative, or neutral perceptions of police. Future research would also benefit from exploring the experiences of therapists and clients who have exclusively relied on non‐police crisis response options to identify the effectiveness and impact of these resources on client care. Addressing these limitations in future research is crucial for an inclusive understanding of safety planning and mental health crisis response.

## 8 Conclusion

The purpose of the study was to better understand therapists' perceptions of police and explore how they describe the experience of navigating police reliance in crisis response. Specifically, we focused on queer therapists' attitudes toward police, given this community's history of police victimization and targeting. Through our analysis, we learned that queer therapists view police as unreliable due, in part, to the perception that police do not have enough crisis response training to effectively deescalate a mental health crisis while also attuning to power dynamics and the social location of the person in distress. Queer therapists drew connections between one's current political climate and the anticipation that police will interact poorly with people who possess fewer social privileges. It is important to add that some participants identified exceptions to the narrative that police were unhelpful or biased when describing instances where police officers de‐escalated a crisis or were able to rely on resources and first aid training that those clinicians did not have. In most instances, however, queer therapists are compiling resources on a case‐by‐case basis when a client is experiencing a mental health crisis, which includes a risk assessment of how helpful or detrimental it would be to involve the police.

Given the limitations identified in this study, future research should prioritize addressing the impact these choices have on therapists from diverse backgrounds and lived experiences who have positive and negative attitudes toward police. Moreover, exploring the outcomes for clients when police were involved or not involved will provide nuanced information regarding the extent to which clients prefer to call 911 or to rely on a non‐police crisis response option. By further exploring these issues, therapists can be more effective in delivering culturally competent care, ultimately reducing the harm that clients in crisis experience.

## Data Availability

The data that support the findings of this study are available from the corresponding author upon reasonable request.
